# Perceptions of Intimate Partner Violence: a cross sectional survey of surgical residents and medical students

**DOI:** 10.5249/jivr.v5i1.147

**Published:** 2013-01

**Authors:** Sheila Sprague, Roopinder Kaloty, Kim Madden, Sonia Dosanjh, Dave J. Mathews, Mohit Bhandari

**Affiliations:** ^a^Department of Clinical Epidemiology and Biostatistics, McMaster University, Canada.; ^*b*^Global Research Solutions, Burlington, Ontario, Canada.; ^*c*^One T, Saint Paul, Minnesota, USA.; ^*d*^Division of Orthopedics Surgery, Department of Surgery, McMaster University, Canada.

**Keywords:** Intimate Partner Violence, Violence prevention, Cross-sectional survey, Medical education

## Abstract

**Background:**

Intimate partner violence (IPV) is an important health issue. Many medical students and residents have received training relating to IPV, but previous studies show that many students feel that their training has been inadequate. Our objective was to assess the knowledge, attitudes and perceptions about IPV among university medical students and surgical residents.

**Methods:**

We administered an online survey to a sample of Ontario medical students and surgical residents. The survey instrument was a modified version of the Provider Survey.

**Results:**

Two hundred medical students and surgical residents participated in the survey (response rate: 29%). Misperceptions about IPV among respondents included the following: 1) victims must get something from the abusive relationships (18.2%), 2) physicians should not interfere with a couple’s conflicts (21%), 3) asking about IPV risks offending patients (45%), 4) Victims choose to be victims (11.1%), 5) it usually takes ‘two to tango’ (18.3%), and 6) some patients’ personalities cause them to be abused (41.1%). The majority of respondents (75.0%) believed identifying IPV was very relevant to clinical practice. The majority of medical students (91.2%) and surgical residents (96.9%) estimated the IPV prevalence in their intended practice to be 10% or less. Most of the medical students (84%) and surgical residents (60%) felt that their level of training on IPV was inadequate and over three quarters of respondents (77.2%) expressed a desire to receive additional education and training on IPV.

**Conclusions:**

There are misconceptions among Canadian medical students and surgical residents about intimate partner violence. These misconceptions may stem from lack of education and personal discomfort with the issue or from other factors such as gender. Curricula in medical schools and surgical training programs should appropriately emphasize educational opportunities in the area of IPV.

## Introduction

Intimate partner violence (IPV) is a serious public health concern that is receiving increasing attention in medical research.^1^The definition includes physical, sexual and/or psychological/ emotional forms of abuse between past or present heterosexual or homosexual partners.^2^Intimate partner violence occurs across all racial, ethnic, regional, and socioeconomic boundaries.^3^ Women are more likely than men to be victims of IPV,^4^ and it is estimated that one in four American women have been victims of IPV in their lifetime.^5^ Richardson et al. found that only 17% of physical abuse victims have ever had it documented in a general practice medical chart, highlighting the serious problem of underreporting of IPV in healthcare.^6^

Intimate partner violence victimization has been reported to impact health and lead to increased use of healthcare services.^7,8^ Intimate partner violence has been linked to mental health disorders such as depression, suicide, and post-traumatic stress disorder.^8^ In a large multinational study by the World Health Organization (WHO), 24 000 women in 10 countries were interviewed about their experiences and beliefs surrounding IPV.^9^ The study found that for all settings combined, women who reported physical violence at least once in their lifetime reported significantly more emotional distress, suicidal thoughts, and suicide attempts than non-abused women.^7^ (Victims of IPV have a 50 to 70% higher chance of having gynecological, central nervous system, and stress-related health problems.^10^ Bonomi et al. found that currently or recently physically abused women have higher total annual health care costs and use more emergency, hospital outpatient, primary care, pharmacy, and specialty services than non-abused women. Mental health service utilization was found to be higher among women abused both physically and non-physically.^10^It is evident that the identification and treatment of IPV victims is highly relevant to healthcare, in which physicians have a key role to play.

A national survey of US medical students identified, 91% of senior students as having had training on IPV, but only one third feeling highly confident in having discussions about IPV with patients.^11^ We conducted a survey with the primary aim of determining medical students’ and surgical residents’ attitudes, beliefs, and perceptions regarding IPV screening, victims, and perpetrators. Secondary aims include examining the level of IPV education/training medical students and surgical residents have received, and exploring how gender and level of education (resident vs. medical student) are related to perceptions of IPV.

## Methods

**Survey Instrument**

Due to the lack of literature on the views and/or knowledge of medical students and surgical residents regarding IPV, we chose to use a modified version of the Provider Survey for our study. The Provider Survey is an instrument intended to measure healthcare providers’ attitudes, beliefs, and self-reported behaviours related to the identification and management of IPV. The Provider Survey is reliable and has been proven valid.^12^Wording modifications were made to the survey to make the questions applicable to medical students and surgical residents. Two versions of the survey were developed; one for medical students and the other for surgical residents. In addition to the Provider Survey, participants were asked to complete questions on their demographics as well as their current perceptions, knowledge and education on IPV. These questions were modified from ones used in recent IPV surveys of medical students, Canadian Orthopaedic Association members and chiropractors.^11,13,14^

There were 23 items in the medical student version of the survey, and 30 items in the surgical resident version. The residents’ survey included questions relevant to their current and previous practice. The medical students’ survey did not include these questions due to their lack of clinical practice experience. Questions were primarily either multiple choices or presented as a series of statements with an associated Likert Scale ranging from strongly disagree to strongly agree. Items were grouped into three categories: 1) demographic information, 2) attitudes, knowledge and education, and 3) clinical relevance of IPV.

**Sampling Frame**

The sampling frame included all medical students and surgical residents currently enrolled at McMaster University, Hamilton, Ontario. We chose to include surgical residents because of our interest in promoting IPV screening in surgical programs. We are unaware of any literature evaluating the attitudes of surgical residents toward IPV. We chose to exclude attending surgeons in our study because the attitudes of attending surgeons have been previously documented.^13^E-mail lists of McMaster University medical students and surgical residents from all years of study were obtained with permission from McMaster University’s Undergraduate Medical Program Office and contacts in the Department of Surgery Residency Programs. Surveys were not sent to students or residents studying outside of McMaster University.

**Survey Administration**

We used Survey Monkey, online survey software, to administer the survey and its cover letter in electronic form. We chose Survey Monkey because it is easy to use for both administrators and participants. Following the initial emailing, three rounds of follow-up emails were sent out to the students. Participants were provided with the opportunity to withdraw at any time.

**Statistical Analysis**

For statistical significance to be reached, 193 trainees were needed for the study sample size. This was based on a population of approximately 700 medical students and surgical trainees at McMaster University, with an error level of 6% and a 95% confidence interval (http://www.custominsight.com/articles/random-sample-calculator.asp).

Survey data were analyzed using PASW version 18.0 (Chicago, IL). Descriptive analyses, including frequency counts and percentages, were performed for all collected data. We conducted Chi-squared tests to determine if there were differences in responses between the surgical residents and medical students using the Contingency Table Calculator.^15^We also conducted a subgroup analysis looking at differences in responses between males and females using chi-squared tests. Surveys with missing data were included in the analysis.

## Results

**Response Rate**

Two hundred trainees responded (29%), meeting the sample size requirements for this study. The response rate for medical students was 23% (127/542) and 49% (73/150) for surgical residents. No information was available about non-respondents, so we are unable to evaluate differences between those who did and did not participate. The survey was administered in the winter term. The lower medical student response rate may be due to possible interference with exam time. Fifteen medical students and eleven surgical residents started the survey but did not complete it. All data collected from incomplete surveys was used in the analysis. There were no withdrawals from the study. 

**Respondent Characteristics**

Respondents ranged in age from 20 to 45 years (mean age = 26± 4.5 years). The majority of the respondents were female (58.3%), which is approximately representative of the population of medical students and surgical residents at McMaster University, and 5% reported a history of IPV (personal history or family history). The top intended specialties for medical students were family medicine (30.8%) and surgery (12.3%). Over two thirds of the surgical residents were specializing in orthopedics (41.1%) or general surgery (27.1%) (Table 1).

**Table:** Table 1:**Respondent Demographics**

Demographic	Overall N (%)	Medical Students N (%)	Surgical Residents N (%)
Mean Age ± Standard Deviation	26.5±4.5	24.5±3.2	30.2±4.2
Gender			
Male	82 (41.4%)	33 (26.2%)	49 (68.1%)
Female	115 (58.1%)	92 (73.0%)	23 (31.9%)
Transgender	1 (0.5%)	1 (0.8%)	0 (0%)
Year			
1	74 (37.9%)	56 (44.1%)	18 (26.5%)
2	60 (30.8%)	47 (37.0%)	13 (19.1%)
3	39 (20.0%)	24 (18.9%)	15 (22.1%)
4	12 (6.2%)	N/A	12 (17.6%)
5	10 (5.1%)	N/A	10 (14.7%)
Intended Medical Specialty for Medical Students			
Family Medicine	40 (30.8%)		
Surgery	16 (12.3%)		
Internal Medicine	13 (10.0%)		
Obstetrics/Gynecology	8 (6.2%)		
Pediatrics	7 (5.4%)		
Psychiatry	6 (4.6%)		
Emergency Medicine	6 (4.6%)		
Anesthesiology	3 (2.3%)		
Neurology	2 (1.5%)		
Unsure	14 (10.8%)		
Other	15 (11.5%)		
Surgical Specialty for Surgical Residents			
Orthopedics	29 (41.4%)		
General Surgery	19 (27.1%)		
Plastic Surgery	7 (10.0%)		
Ophthalmology	4 (5.7%)		
Urology	4 (5.7%)		
Neurosurgery	3 (4.3%)		
Pediatric General Surgery	2 (2.9%)		
Cardiac Surgery	1 (1.4%)		
Otolaryngology/Head/Neck Surgery	1 (1.4%)		

Totals may not add to 200 participants due to missing data.

**Misperceptions about IPV**

**Figure 1: Medical Students' and Surgical Residents' Estimated Prevalence of IPV in their Practice:**
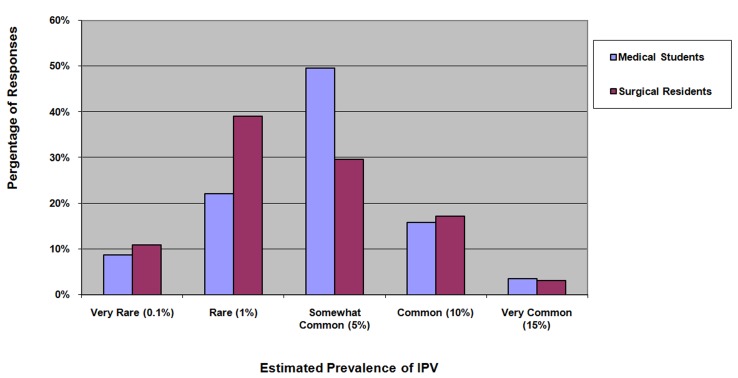


Most respondents (91.2% of medical students and 96.9% of surgical residents) estimated the IPV prevalence in their intended practice to be 10% or less (Figure 1).Respondents held misperceptions about the following issues: 1) victims must get something from the abusive relationships (18.2%), 2) physicians should not interfere with a couple’s conflicts (21%), 3) asking about IPV risk offending patients (45%), 4) victims choose to be victims (11.1%), 5) it usually takes ‘two to tango’ (18.3%), and 6) some patients’ personalities cause them to be abused (41.1%) **(Appendix A)**. 

Surgical residents were significantly more likely to hold misperceptions about a victim’s role in their abuse (‘it takes two to tango’) compared to medical students (28.2% vs. 12.9%, p=0.038) (Table 2). Males were significantly less likely to disagree with victim-blaming statements than females, such as “People are only victims if they choose to be” (77.8% vs. 96.2% strongly disagree/disagree, p<0.001) and “Women who choose to step out of traditional roles are a major cause of IPV” (75.3% vs. 93.4% strongly disagree/disagree, p=0.001) (Table 3).Many respondents were concerned for their personal safety when asking a patient battering (32.6% strongly agree/agree), and nearly one quarter of respondents fear that they will offend patients if they ask about IPV (22.8% strongly agree/agree) (Table 2).Many respondents has misconceptions about batterers, for example, the abuse would stop if the batterer stopped using alcohol (34.9% agree/strongly agree) **(Appendix B)**.

**Table:** Table 2:**General Knowledge, Personal Comfort and Attitudes**

	Overall N (%)	Medical Students N (%)	Surgical Residents N (%)	P Value*
I am (or would be) afraid of offending the patient if I ask about IPV.				0.082
Strongly agree/ agree	41 (22.8%)	32 (27.6%)	9 (14%)	
Neutral	40 (22.2%)	22 (19.0%)	18 (28.1%)	
Disagree/Strongly Disagree	99 (55.0%)	62 (53.5%)	37 (57.8%)	
I don’t know how to ask about the possibility of IPV.				<0.001**
Strongly agree/ agree	50 (27.8%)	44 (37.6%)	6 (9.5%)	
Neutral	42 (23.3%)	25 (21.4%)	17 (27.0%)	
Disagree/Strongly Disagree	88 (48.9%)	48 (41%)	40 (63.4%)	
When it comes to domestic violence, it usually “takes two to tango.”				0.033**
Strongly agree/ agree	7 (3.9%)	3 (2.6%)	4 (6.3%)	
Neutral	26 (14.4%)	12 (10.3%)	14 (21.9%)	
Disagree/Strongly Disagree	148 (81.8%)	102 (87.2%)	46 (71.9%)	
I am (or would be) reluctant to ask batterers about their abusive behavior out of concern for my personal safety.				0.497
Strongly agree/ agree	57 (32.6%)	38 (34.0%)	19 (30.2%)	
Neutral	42 (24.0%)	29 (25.9%)	13 (20.6%)	
Disagree/Strongly Disagree	76 (43.4%)	45 (40.2%)	31 (49.2%)	
I am (or would be) afraid of offending patients if I ask about their abusive behavior.				0.012
Strongly agree/ agree	78 (44.6%)	59 (52.7%)	19 (30.1%)	
Neutral	37 (21.1%)	22 (19.6%)	15 (23.8%)	
Disagree/Strongly Disagree	60 (34.3%)	31 (27.7%)	29 (46.1%)	
I am afraid that talking to the batterer will increase risk for the victim.				0.009
Strongly agree/ agree	107 (61.1%)	78 (69.6%)	29 (46.1%)	
Neutral	44 (25.1%)	22 (19.6%)	22 (34.9%)	
Disagree/Strongly Disagree	24 (13.7%)	12 (10.7%)	12 (19.1%)	
I feel I can effectively discuss issues of battering and abuse with a battering patient.				0.016
Strongly agree/ agree	19 (10.9%)	10 (8.9%)	9 (14.3%)	
Neutral	48 (27.4%)	24 (21.4%)	24 (38.1%)	
Disagree/Strongly Disagree	108 (61.7%)	78 (69.7%)	30 (47.6%)	
Time constraints				0.004
Strongly agree/ agree	160 (82.9%)	111 (89.5%)	49 (71.0%)	
Neutral	16 (8.3%)	7 (5.6%)	9 (13.0%)	
Disagree/Strongly Disagree	17 (8.8%)	6 (4.8%)	11 (15.9%)	
Lack of knowledge of what to ask				0.010
Strongly agree/ agree	129 (66.8%)	91 (73.4%)	38 (55.1%)	
Neutral	29 (15.0%)	18 (14.5%)	11 (15.9%)	
Disagree/Strongly Disagree	35 (18.1%)	15 (12.1%)	20 (28.9%)	
Lack of knowledge of what to do if patient says “yes” to inquiry				<0.001
Strongly agree/ agree	121 (62.7%)	90 (72.5%)	31 (44.9%)	
Neutral	26 (13.5%)	17 (13.7%)	9 (13%)	
Disagree/Strongly Disagree	46 (23.8%)	17 (13.7%)	29 (42%)	
Personal discomfort with the issue				0.003
Strongly agree/ agree	102 (52.8%)	77 (62.1%)	25 (36.2%)	
Neutral	37 (19.2%)	19 (15.3%)	18 (26.1%)	
Disagree/Strongly Disagree	54 (28.0%)	28 (22.6%)	26 (37.6%)	
Lack of knowledge of community resources				0.754
Strongly agree/ agree	135 (69.9%)	88 (71.0%)	47 (68.1%)	
Neutral	32 (16.6%)	21 (16.9%)	11 (15.9%)	
Disagree/Strongly Disagree	26 (13.5%)	15 (12.1%)	11 (15.9%)	

* Chi-Squared test ** Fisher’s exact test (Has an expected value of less than 5). Totals may not add to 200 participants due to missing data.

**Table:** Table 3:**Responses of Male versus Female Respondents**

Question	Males N (%)	Females N (%)	P Value*
History of IPV	2 (2.4%)	8 (7.0%)	
Personal	0 (0%)	5 (62.5%)	
Family	2 (100%)	3 (37.5%)	
Amount of IPV training/education received			0.045
None	28 (34.6%)	54 (49.1%)	
Any	53 (65.4%)	56 (50.9%)	
It is demeaning to patients to question them about abuse.			0.051**
Strongly agree/ agree	3 (4.2%)	2 (1.9%)	
Neutral	15 (20.5%)	10 (9.4%)	
Disagree/Strongly Disagree	54 (75.0%)	94 (88.7%)	
People are only victims if they choose to be.			<0.001**
Strongly agree/ agree	6 (8.3%)	0 (0%)	
Neutral	10 (13.9%)	4 (3.8%)	
Disagree/Strongly Disagree	56 (77.8%)	102 (96.2%)	
Women who choose to step out of traditional roles are a major cause of IPV.			<0.001**
Strongly agree/ agree	3 (4.2%)	0 (0%)	
Neutral	15 (20.5%)	7 (6.6%)	
Disagree/Strongly Disagree	54 (75.3%)	99 (93.4%)	

* Chi-Squared test **Has an expected value of less than 5 Totals may not add to 200 participants due to missing data.

**Barriers to Assessment of IPV**

Key barriers to IPV assessment perceived by respondents included lack of time (82.9%), lack of knowledge of what to ask (66.8%), lack of knowledge of community resources (69.9%), and personal discomfort (52.8%) (Table 2). Medical students were significantly more likely to have issues with lack of time (89.5% vs. 71.0%, p=0.004), lack of knowledge of what to say (73.4% vs. 55.1%, p=0.010), lack of knowledge of what to do if a patient is abused (72.5% vs. 44.9%, p<0.001), and personal discomfort (62.1% vs. 36.2%, p=0.003) when compared to surgical residents (Table 2).42% of respondents reported that the main barrier to screening for IPV is a lack of training **(Appendix C).**

**Need for Education and Training**

Most respondents identified IPV identification as relevant in their practice (89%) but many were unsure or incorrect about legal reporting requirements (20.0% unsure, 12.1% incorrect) (Table 4). Almost all respondents had little or no previous IPV training (99%), yet only 75% believed their education was inadequate. Most trainees supported additional training and educational initiatives in IPV (77.2%), and 42% cited their lack of education and training as the primary barrier to routine assessment (Table 4). Medical students were significantly more likely to report a lack of training (83.9% vs. 60.3%, p=0.001) and desire for increased IPV education (87.9% vs. 58.0%, p=0.062). 

**Table:** Table 4:**Relevance and Education**

		Overall N (%)	Medical Students N (%)	Surgical Residents N (%)	P Value*
Relevance of identifying IPV victims in practice	Not at all relevant	2 (1.0%)	1 (0.8%)	1 (1.5%)	NA
	Possibly relevant	15 (7.8%)	11 (8.9%)	4 (5.9%)	
	Somewhat relevant	27 (14.0%)	18 (14.5%)	9 (13.2%)	
	Very relevant	144 (75.0%)	94 (75.8%)	50 (73.5%)	
	Depends on Specialty	4 (2.1%)	----***	4 (5.9%)	
Level of comfort asking a woman about abuse	Very uncomfortable	4 (2.1%)	1 (0.8%)	3 (4.3%)	0.023**
	Uncomfortable	31 (16.1%)	24 (19.4%)	7 (10.1%)	
	Somewhat comfortable	91 (47.2%)	64 (51.6%)	27(39.1%)	
	Comfortable	57 (29.5%)	29 (23.4%)	28 (40.6%)	
	Very comfortable	10 (5.2%)	6 (4.8%)	4 (5.8%)	
Is health care provider reporting of intimate partner violence mandatory in Canada?	Yes	23 (12.1%)	16 (12.9%)	7 (10.6%)	0.877
	No	129 (67.9%)	84 (67.7%)	45 (68.2%)	
	Unsure	38 (20.0%)	24 (19.4%)	14 (21.2%)	
Amount of IPV education/training received	None	83 (43.0%)	66 (53.2%)	17 (24.6%)	<0.001**
	Some	108 (56.0%)	56 (45.2%)	52 (75.4%)	
	Extensive	2 (1.0%)	2 (1.6%)	0 (0.0%)	
Adequate amount of IPV training received thus far	Yes	21 (10.9%)	8 (6.5%)	13 (19.1%)	0.001
	No	145 (75.5%)	104 (83.9%)	41 (60.3%)	
	Unsure	26 (13.5%)	12 (9.7%)	14 (20.6%)	
Desire for additional training on the assessment and treatment of IPV	Yes	149 (77.2%)	109 (87.9%)	40 (58.0%)	<0.001**
	No	16 (8.3%)	4 (3.2%)	12 (17.4%)	
	Unsure	22 (11.4%)	7 (5.6%)	15 (21.7%)	
	Not relevant to my intended practice	6 (3.1%)	4 (3.2%)	2 (2.9%)	
Providing medical students with more education/training on intimate partner violence would help increase the number of physicians that screen for it	Strongly agree	47 (24.4%) 110	28 (22.6%)	19 (27.5%)	0.032**
	Agree	(57.0%)	79 (63.7%)	31 (44.9%)	
	Unsure	26 (13.5%)	11 (8.9%)	15 (21.7%)	
	Disagree	10 (5.2%)	6 (4.8%)	4 (5.8%)	
	Strongly Disagree	0 (0.0%)	0 (0.0%)	0 (0.0%)	

* Chi-Squared test ** Has an expected value of less than 5 Totals may not add to 200 participants due to missing data.

**Clinical Assessment of IPV**

When considering the last few months of clinical practice, trainees reported seldom or rarely asking patients about IPV with injuries (66.1%), pelvic pain (33.8%), irritable bowel syndrome (45.1%), headaches (46.8%), depression/anxiety (33.9%), or high blood pressure (59%) **(Appendix D)**.

Many surgical residents (48.4%) reported that they had identified a victim of IPV and 27.4% of residents reported that they had identified a batterer. Only 9.7% of surgical residents reported that their clinical setting has guidelines for detecting and managing IPV, and over one quarter (25.8%) were unsure if there are guidelines in their clinical setting **(Appendix D)**.

## Discussion

In this survey of 200 Canadian medical students and surgical residents, our findings suggest that medical students and surgical residents have multiple misperceptions about IPV and have not received adequate training on the identification and treatment of IPV. Most respondents underestimated the IPV prevalence in their intended practice. Despite having misconceptions about IPV and underestimating its prevalence, the majority of respondents believed identifying IPV was very relevant to clinical practice. In addition, most of the respondents acknowledged that their level of training on IPV was inadequate and over three quarters of respondents would like to receive additional education and training on IPV. 

Medical student and surgical trainees incorrectly believed that the prevalence of IPV is 10% or less. These results are similar to the findings of two previous surveys of health care practitioners. In a recent survey of orthopaedic surgeons, most respondents indicated that the prevalence of IPV in their practice was rare (<1%).^13^ In a similar study of Ontario chiropractors, the majority of respondents indicated that the prevalence of IPV in their practice was between 0.1% and 1%.^14^ In contrast to the survey findings, multiple prevalence studies have shown that the lifetime prevalence of IPV is much higher. For example, several American, Australian, and Canadian studies have found that IPV prevalence is well over 10% in both emergency medicine^16-20^ and family medicine.^21-25^ In addition, the PRAISE Investigators^26^ recently found that the 12 month prevalence of IPV in orthopaedic fracture clinics, one of the most common specialties among our respondents, was over 30%.

Almost half of the respondents held the misconception that patients would be offended if they were asked about IPV. This finding is similar to a survey of Ontario chiropractors, which shows that almost half of chiropractors were afraid of offending patients when asking about IPV.^14^ This finding contradicts other research by Hurley et al.^27^ who found that 86% of men and women presenting to Canadian emergency departments agreed that health care providers should screen for IPV. Similarly, Caralis et al.^28^ established that the majority of American survey respondents believe doctors should screen for abuse in their practices. In addition, Feder et al.^29^ conducted a meta-analysis that showed women who have been abused support screening programs for IPV in a health care setting. Dispelling the misconception that women do not wish to be screened for IPV and providing additional education on IPV could help ensure additional screening for IPV among future health care practitioners.

Education in IPV was valued among respondents; however, most reported feeling that they have received an inadequate level of education and training on IPV and have a desire to receive additional training on the assessment and treatment of IPV. Both of these results were higher for medical students, which we speculate is attributed to their shorter time in the medical curriculum. These results are similar to a survey of American medical students that concluded that despite national interest in IPV issues, efforts in U.S. medical schools to increase IPV screening and prevention have not achieved saturation.^11^ Similarly, a recent report from the Association of American Medical Colleges found that 20% of U.S. graduating physicians in 2004 believed that the curriculum time dedicated to IPV was inadequate.^30^ Hamberger^31^ has suggested that, although most medical schools educate students on IPV in some form, the teaching is mainly done in a basic science module as opposed to in a clinical setting. Edwardsen et al.^32^ have showed that a structured IPV training program with use of mnemonics and clinical role-playing can help medical students to ask their patients questions about their history of IPV. In addition, Chapin et al. revealed that emergency medical personnel who received IPV training from a domestic violence center were better informed about IPV services and the obstacles faced by victims.^33^ One clinic-based IPV education program for pediatric residents has increased IPV screening from less than 1% to over 30% 8 months after program completion.^34^ We suggest that IPV education be included in both medical and residency training and that it should focus on clinic-based practical exercises.

Nearly one third of respondents were either incorrect or unsure when asked if health care provider reporting of IPV is mandatory in Canada. Reporting of IPV is not mandatory in Canada,^34^ but it is mandatory in some American states.^35^ Most surgical residents reported screening for IPV only seldom or never, which is consistent with the findings of a recent survey of orthopaedic surgeons^13^ and of U.S. medical residents.^11^ In addition, it was very uncommon for surgical residents to screen patients with illnesses linked to IPV such as hypertension/ coronary artery disease, irritable bowel syndrome, or headaches, indicating a need for additional education on the identification and screening of IPV. The survey found that residents were more likely to screen patients with injuries for IPV. This is consistent with a study of U.S. residents^36^ and of primary care physicians.^37^

Medical students were more likely to report that they felt time constraints, lack of knowledge, and personal discomfort compared to residents. Residents may have had more opportunities to come across abused women due to increased time in clinical settings compared to medical students. Perhaps some of the residents’ knowledge comes not from formal academic training, as in medical school, but from experiential learning in a clinical setting. 

In previous studies, IPV training has been proven successful in raising awareness as well as improving the ability of healthcare professionals to detect IPV. Warburton et al.^38^ reported that a brief IPV educational program improved dental hospital staff’s attitudes and knowledge about IPV. As well, an educational program for internal medicine residents was both well received and effective at improving detection of IPV victims.^39^ Three key questions aimed at raising suspicion of IPV were included in a questionnaire; 54% of the intervention group were able to answer at least two out of three questions correctly compared to 20% of the control group.^39^

This study has provided valuable insight into the knowledge, education, attitudes and perceptions of medical students and surgical residents regarding the topic of IPV. One of the strengths of the study is the survey instrument, which was created by domestic violence experts and validated in IPV surveys of health care providers. It has met basic face and basic content validity, although we did use a slightly modified version of the Provider Survey for students and residents which have not been validated. The survey also had an adequate sample size. However, the study also had some limitations. Our survey had a relatively low response rate, which may be a potential source of bias. It is possible that responders differed in some characteristics compared to non-responders. For example, non-responders could be more likely than responders to have experienced IPV, which would influence many of the results. Our survey was restricted to only medical students and surgical residents from McMaster University who could be contacted via email. It remains unclear whether our findings are generalizable to other universities and jurisdictions. This study was a descriptive, cross-sectional study that can only be used to identify perceptions and barriers regarding IPV inquiry. Causal inferences about specific variables and outcomes cannot be made.

## Conclusion

Misconceptions exist among one Canadian medical school’s medical students and surgical residents about IPV and may be related to lack of education and low self-efficacy, or possibly to other factors such as gender. Curricula in medical schools and surgical training programs should appropriately emphasize educational opportunities in the area of IPV. Future research should explore the most optimal methods of disseminating IPV information among students and health care providers in order to increase awareness of IPV and reduce these misconceptions. It is anticipated that increased awareness of IPV among health care providers will motivate them to seek additional IPV knowledge and training, and ultimately increase screening and care of patients experiencing IPV in their practice.
